# Crystal structure of an aceto­nitrile solvate of 2-(3,4,5-triphen­ylphen­yl)acetic acid

**DOI:** 10.1107/S2056989024009976

**Published:** 2024-10-24

**Authors:** Pierre Seidel, Franziska Gottwald, Eric Meier, Monika Mazik

**Affiliations:** a Institut für Organische Chemie Technische Universität Bergakademie, Freiberg, Leipziger Str 29 09599 Freiberg/Sachsen Germany; Texas A & M University, USA

**Keywords:** carb­oxy­lic acid dimers, C—H⋯π inter­actions, aceto­nitrile solvate, crystal structure

## Abstract

Crystal growth of 2-(3,4,5-triphen­ylphen­yl)acetic acid (**1**) from aceto­nitrile yields a monosolvate, C_26_H_20_O_2_·CH_3_CN, of the space group *P*1. In the crystal, the title mol­ecule adopts a conformation in which the three phenyl rings are arranged in a paddlewheel-like fashion around the central arene ring and the carboxyl residue is oriented nearly perpendicular to the plane of this benzene ring. Inversion-symmetric dimers of O—H⋯O-bonded mol­ecules of **1** represent the basic supra­molecular entities of the crystal structure. These dimeric mol­ecular units are further linked by C—H⋯O=C bonds to form one-dimensional supra­molecular aggregates running along the crystallographic [111] direction. Weak C_ar­yl_—H⋯N inter­actions occur between the mol­ecules of **1** and aceto­nitrile.

## Abstract 2

The title mol­ecule adopts a conformation in which the three phenyl rings are arranged in a paddlewheel-like fashion around the central arene ring and the carboxyl residue is oriented nearly perpendicular to the plane of this benzene ring. Inversion-symmetric dimers of O—H⋯O-bonded mol­ecules represent the basic supra­molecular entities of the crystal structure. These dimeric mol­ecular units are further linked by C—H⋯O=C bonds, forming one-dimensional supra­molecular aggregates along [111].

## 1. Chemical context

Phenyl­acetic acid (PAA) and its derivatives have a wide range of biological activities (Cook, 2019 ▸; Jiao *et al.*, 2022 ▸; Perez *et al.*, 2023 ▸). It is important to note that this class of compounds has played an important role in the development of numerous drugs, for example as a building block of drug mol­ecules or as a starting material for their syntheses (Treves & Testa, 1952 ▸; Vardanyan & Hruby, 2006 ▸). Examples include drugs such as diclofenac, ibuprofen, flurbiprofen, cyclo­pentolate and atenolol. They have a wide range of uses, including non-steroidal anti-inflammatory drugs, analgesics, anti­cancer agents, mydriatics and cyclo­plegics, among others. Compounds bearing one or two phenyl substituents on the benzene ring of PAA have been reported to have anti-tumor activity (Lade *et al.*, 2023 ▸) and some have been proposed as candidates for the treatment of Alzheimer’s disease (Wilson *et al.*, 2015 ▸). The synthesis of new derivatives of phenyl­acetic acid is of great importance due to their inter­esting properties and the possibility of their wide application.
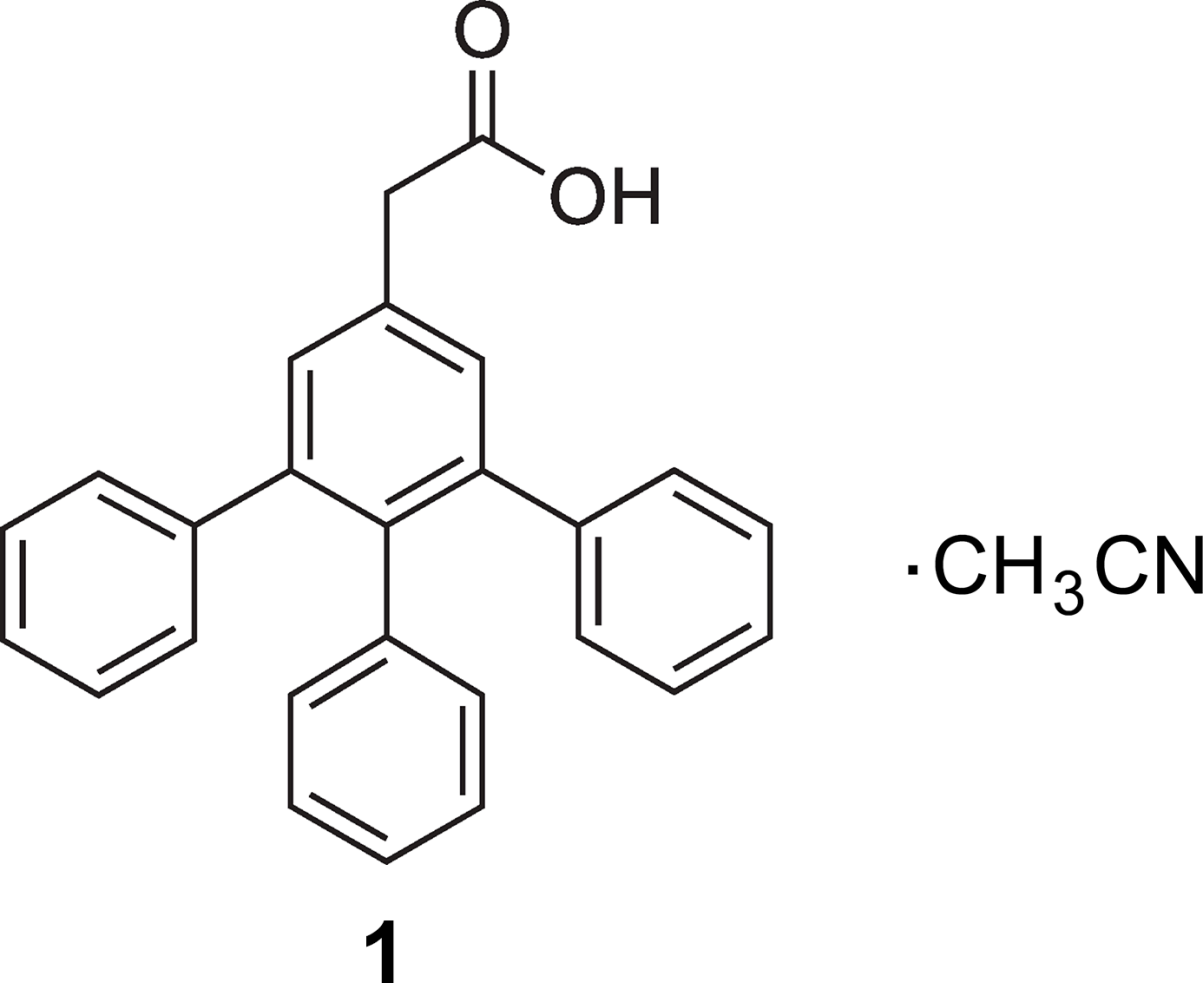


The title compound, which bears three phenyl substituents in positions 3, 4 and 5 of the benzene ring of PAA, has been prepared by us as a compound with potentially valuable biological activities and with the ability to act as a starting material for various functionalizations (Mazik & Seidel, 2024 ▸). Crystallization of this compound from aceto­nitrile yielded a solvate, the crystal structure of which is described in this article.

## 2. Structural commentary

**Figure 1:**
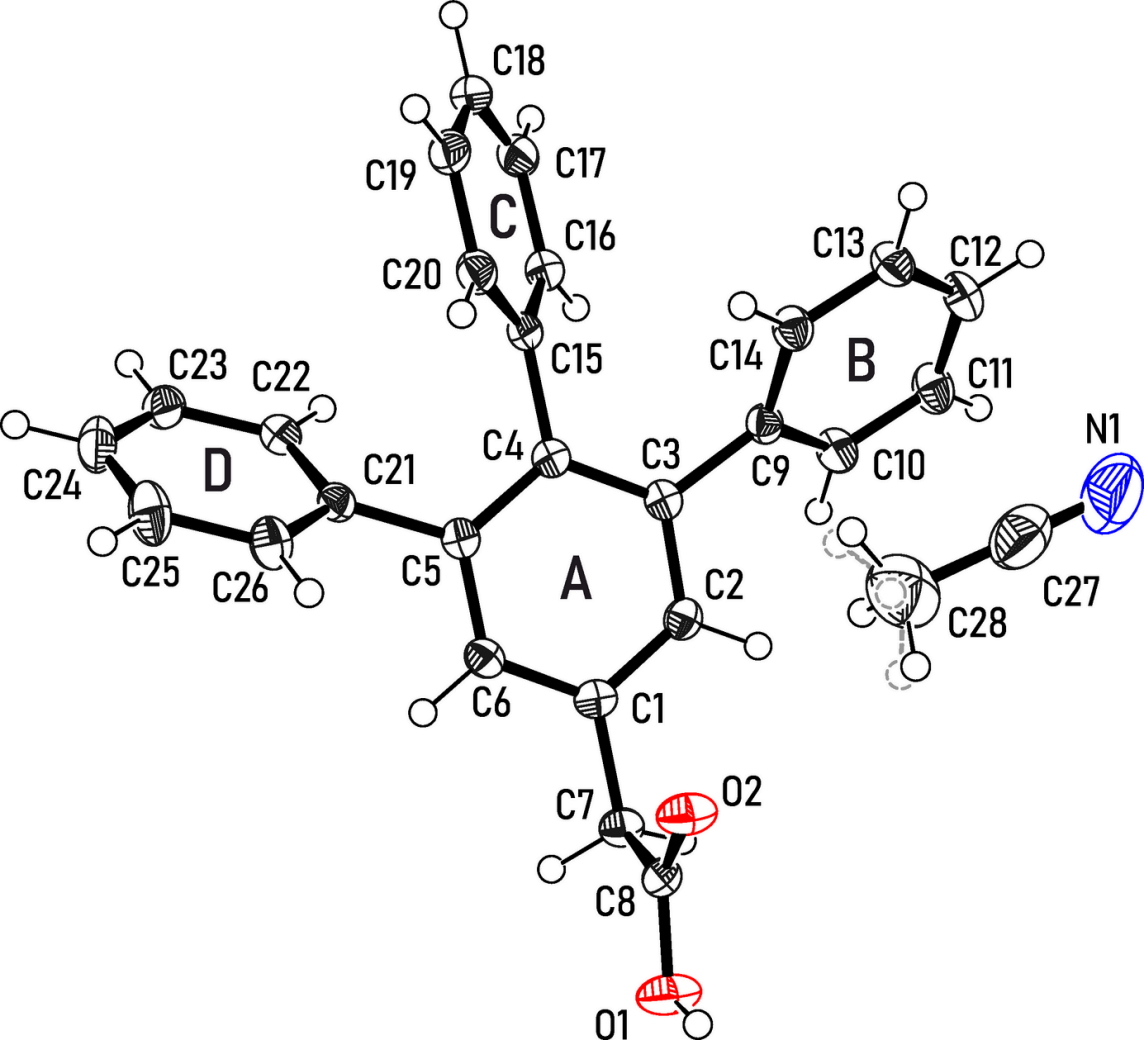
Perspective view of the mol­ecular structure of **1**·CH_3_CN including the atom labeling and ring specification. Atomic displacement ellipsoids are drawn at the 50% probability level. Broken gray lines indicate the second position of the disordered methyl hydrogen atoms of the solvent.

The title compound 2-(3,4,5-triphen­ylphen­yl)acetic acid (**1**) forms a solvate with aceto­nitrile, which crystallizes in the space group *P*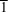 and contains one formula unit of each mol­ecular species within its asymmetric unit (see Fig. 1 ▸). A slight disorder of the solvent is observed, as its methyl hydrogen atoms occupy two positions in a roughly 50:50 distribution. The three phenyl substituents attached to the central benzene ring of **1** (*A*, C1–C6) uniformly adopt a tilted orientation with respect to the plane of this ring, resulting in a mol­ecular geometry that resembles a paddlewheel. The inclination angles of the aromatic planes in relation to the central ring (*A*) amount to 46.39 (6)° (ring *B*, C9–C14), 59.72 (6)° (ring *C*, C15–C20) and 56.17 (6)° (ring *D*, C21–C26), respectively. The plane through the carboxyl group of the mol­ecular side arm is oriented nearly perpendicular [84.9 (1)°] with respect to the central arene ring.

## 3. Supra­molecular features

**Figure 2:**
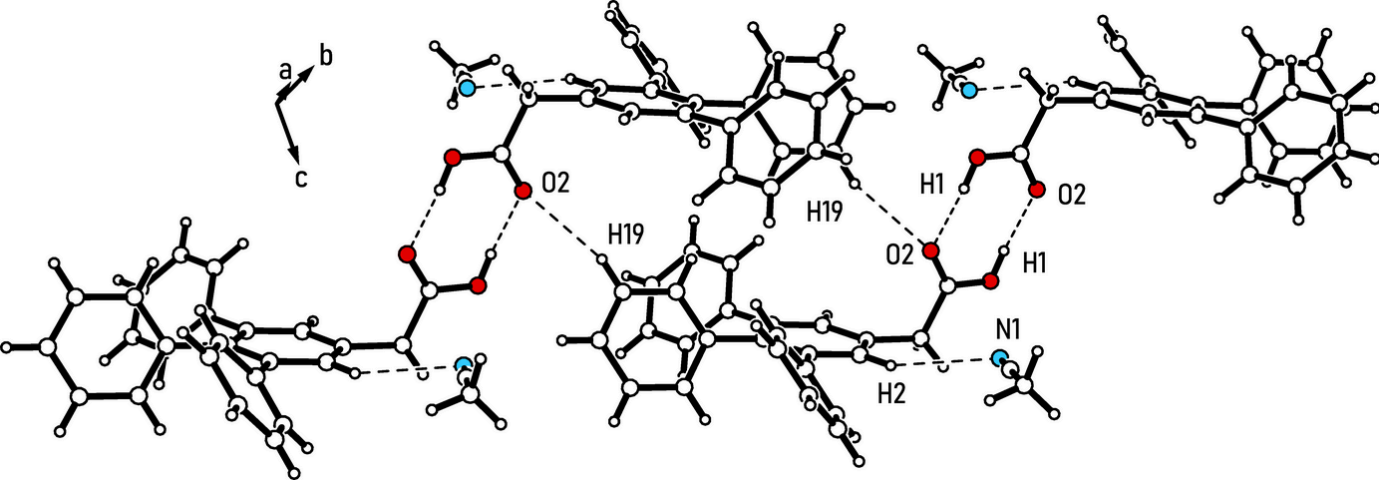
Excerpt of the chain-like supra­molecular aggregates with labeling of atoms involved in O—H⋯O and C—H⋯O/N hydrogen bonding. Color code: red – O; blue – N. Only the major disorder component of the solvent mol­ecules is depicted.

**Figure 3:**
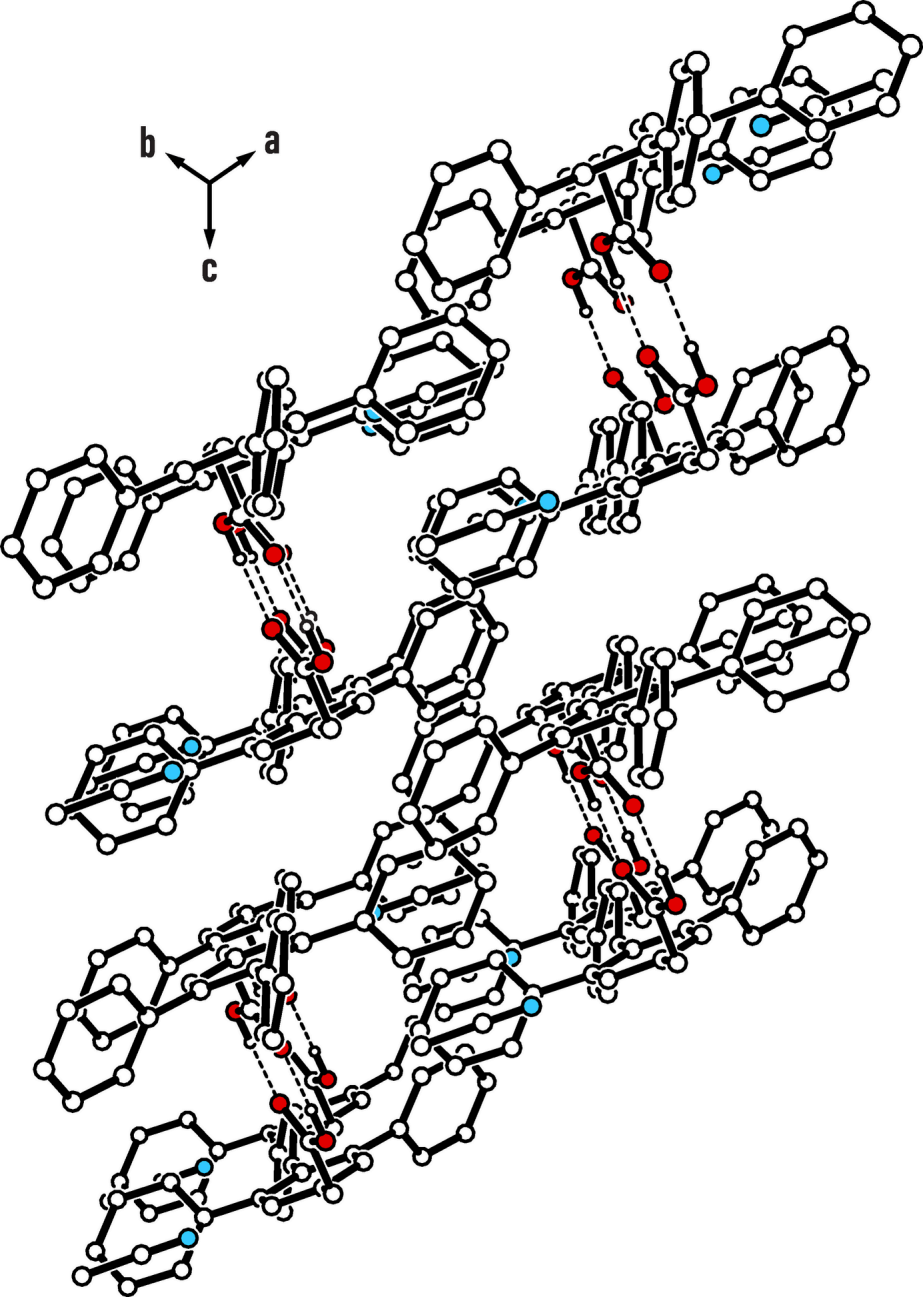
Packing diagram of the structure viewed along the [111] direction (parallel to the propagation of the supra­molecular chains). Hydrogen atoms excluded from hydrogen bonding are omitted for clarity. Dashed lines symbolize the cyclic hydrogen-bond motif between carboxyl functionalities. Color code: red – O; blue – N.

The most dominant non-covalent inter­actions within the crystal structure (see Fig. 2 ▸ and Table 1 ▸) are classical hydrogen bonds between the carboxyl moieties of the inversion-related mol­ecules [*d*(H1⋯O2) = 1.62 (2) Å, O—H⋯O = 174 (2)°], forming a cyclic synthon of the graph set 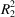
(8) (Etter *et al.* 1990 ▸; Bernstein *et al.*, 1995 ▸; for a discussion on supra­molecular synthons in crystal engineering, including those formed by carboxyl groups, see: Desiraju, 1995 ▸). As shown in Fig. 3 ▸, these dimers are connected along the [111] direction by pairs of C—H⋯O bonds involving the aryl hydrogen atom H19 and the carbonyl oxygen atom O2 [*d* = 2.56 Å, C—H⋯O = 163°; for other examples of C—H⋯O bonds, see: Desiraju & Steiner, 1999 ▸; Desiraju, 2005 ▸; Mazik *et al.*, 1999*a* ▸, 2005 ▸, 2010 ▸; Ebersbach *et al.*, 2023 ▸]. Consequently, O2 acts as a bifurcated binding site for hydrogen bonding. The solvent mol­ecule appears to be fixed in its position by a weak C—H⋯N bond involving the atom H2 of the central arene ring [*d* = 2.71 Å, C—H⋯N = 150°; for other examples of C—H⋯N bonds, see: Reddy *et al.*, 1996 ▸; Desiraju & Steiner, 1999 ▸; Thalladi *et al.*, 2000*a* ▸,*b* ▸; Mazik *et al.*, 1999*b* ▸, 2000*a* ▸,*b* ▸, 2001 ▸, 2005 ▸]. Since the peripheries of the one-dimensional supra­molecular aggregates are formed by the non-polar units of the host mol­ecules, van der Waals forces contribute significantly to the cohesion of the crystal structure. Moreover, multiple short distances between C—H units and aromatic moieties suggest the presence of C—H⋯π inter­actions (Nishio *et al.*, 2009 ▸, 2011 ▸).

## 4. Database survey

Based on the search in the Cambridge Structural Database (CSD, Version 5.45, update June 2024; Groom *et al.*, 2016 ▸) for phenyl­acetic acid and its derivatives with one to five phenyl substituents on the benzene ring, the crystal structures of phenyl­acetic acid (ZZZMLY01; Hodgson & Asplund, 1991 ▸) and 4-bi­phenyl­acetic acid (KUZWEI; Van Eerdenbrugh *et al.*, 2010 ▸) were found. The latter compound is a potent non-steroidal anti-inflammatory drug (felbinac; Hosie & Bird, 1994 ▸). Furthermore, the crystal structures of the complexes of phenyl­acetic acid with its potassium salt (KHDPAC; Bacon & Curry, 1960 ▸), benzamide (MECHAF; Chaudhari & Suryaprakash, 2012 ▸) and hexa­methyl­ene­tetra­mine (urotropine) (VIJTIR; Mak *et al.*, 1986 ▸) are reported. Single crystal structures of complexes of felbinac with tryptamine and 1,2-di­phenyl­ethyl­enedi­amine are also described (JOZMEQ, Koshima *et al.*, 1998 ▸; EDOLAL, Imai *et al.*, 2007 ▸). Similar to **1**, in the solvent-free crystal structures with the reference codes ZZZMLY01 and KUZWEI, the carb­oxy groups of the adjacent mol­ecules form the dimer synthon (Desiraju, 1995 ▸). Furthermore, the aromatic cores are linked *via* edge-to-face C—H⋯π inter­actions.

## 5. Synthesis and crystallization

Compound **1** was prepared as previously described (Mazik & Seidel, 2024 ▸). Crystallization was carried out from aceto­nitrile by slow evaporation of the solvent. Crystal habit: colorless, rhombic plates.

## 6. Refinement

Crystal data, data collection and structure refinement details are summarized in Table 2 ▸. All non-hydrogen atoms were refined anisotropically. The hydrogen atom of the carboxyl group (H1) was located in a difference-Fourier map and refined freely. The remaining hydrogen atoms were positioned geometrically and refined isotropically using a riding model, with C—H bond distances of 0.95 Å (arene), 0.98 Å (meth­yl) and 0.99 Å (methyl­ene). Additionally, their thermal displacement ellipsoids [*U*_iso_(H)] were set to 1.2 *U*_eq_(C) and 1.5 *U*_eq_(C) for arene/methyl­ene and methyl groups, respectively.

## Supplementary Material

Crystal structure: contains datablock(s) I. DOI: 10.1107/S2056989024009976/jy2053sup1.cif

Structure factors: contains datablock(s) I. DOI: 10.1107/S2056989024009976/jy2053Isup2.hkl

Supporting information file. DOI: 10.1107/S2056989024009976/jy2053Isup3.cml

CCDC reference: 2391130

Additional supporting information:  crystallographic information; 3D view; checkCIF report

## Figures and Tables

**Table 1:** Geometric data (Å, °) of short inter­molecular inter­actions *Cg*(*A*) and *Cg*(*D*) refer to the centers of gravity of the rings C1–C6 and C21–C26, respectively

*D*—H⋯*A*/*Cg*	*D*—H	H⋯*A*/*Cg*	*D*⋯*A*/*Cg*	*D*—H⋯*A*/*Cg*
O1—H1⋯O2^i^	1.01 (2)	1.62 (2)	2.624 (1)	174 (2)
C2—H2⋯N1^ii^	0.95	2.71	3.567 (2)	150
C19—H19⋯O2^iii^	0.95	2.56	3.477 (1)	163
C7—H7*B*⋯*Cg*(*D*)^iv^	0.99	2.97	3.746 (1)	136
C10—H10⋯*Cg*(*A*)^v^	0.95	2.97	3.509 (1)	119

Symmetry codes: (i) −*x*, −*y*, −*z* + 1; (ii) −*x* + 1, −*y*, −*z* + 1; (iii) −*x* + 1, −*y* + 1, −*z* + 2; (iv) −*x*, −*y* + 1, −*z* + 1; (v) −*x* + 1, −*y* + 1, −*z* + 1.

**Table 2:** Experimental details

Crystal data	
Chemical formula	C_26_H_20_O_2_·C_2_H_3_N
*M*_r_	405.47
Crystal system, space group	Triclinic, *P* 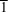
Temperature (K)	123
*a*, *b*, *c* (Å)	10.0737 (5), 11.1039 (6), 11.5835 (6)
α, β, γ (°)	107.025 (4), 114.263 (4), 93.898 (4)
*V* (Å^3^)	1102.82 (11)
*Z*	2
Radiation type	Mo *K*α
μ (mm^−1^)	0.08
Crystal size (mm)	0.32 × 0.23 × 0.12

Data collection	
Diffractometer	Stoe Stadivari
Absorption correction	–
No. of measured, independent and observed [*I* > 2σ(*I*)] reflections	29260, 5839, 4519
*R*_int_	0.029
(sin θ/λ)_max_ (Å^−1^)	0.682

Refinement	
*R*[*F*^2^ > 2σ(*F*^2^)], *wR*(*F*^2^), *S*	0.040, 0.106, 1.07
No. of reflections	5839
No. of parameters	286
H-atom treatment	H atoms treated by a mixture of independent and constrained refinement
Δρ_max_, Δρ_min_ (e Å^−3^)	0.29, −0.23

Computer programs: *X-AREA*, *X-AREA Recipe*, *X-RED32* and *LANA* (Stoe & Cie, 2002 ▸), *SHELXT2018/2* (Sheldrick, 2015*a* ▸), *SHELXL2019/3* (Sheldrick, 2015*b* ▸), *OLEX2* (Dolomanov *et al.*, 2009 ▸), *XP* in *SHELXTL* (Sheldrick, 2008 ▸) and *ORTEP-3 for Windows* (Farrugia, 2012 ▸).

## supplementary crystallographic information

### 2-(3,4,5-Triphenylphenyl)acetic acid acetonitrile monosolvate Crystal data

**Table:**

C_26_H_20_O_2_·C_2_H_3_N	*Z* = 2
*M**_r_* = 405.47	*F*(000) = 428
Triclinic, *P*1	*D*_x_ = 1.221 Mg m^−^^3^
*a* = 10.0737 (5) Å	Mo *K*α radiation, λ = 0.71073 Å
*b* = 11.1039 (6) Å	Cell parameters from 20520 reflections
*c* = 11.5835 (6) Å	θ = 2.0–31.2°
α = 107.025 (4)°	µ = 0.08 mm^−^^1^
β = 114.263 (4)°	*T* = 123 K
γ = 93.898 (4)°	Irregular, colorless
*V* = 1102.82 (11) Å^3^	0.32 × 0.23 × 0.12 mm

### 2-(3,4,5-Triphenylphenyl)acetic acid acetonitrile monosolvate Data collection

**Table:**

Stoe Stadivari diffractometer	4519 reflections with *I* > 2σ(*I*)
Radiation source: Primux 50 Mo	*R*_int_ = 0.029
Graded multilayer mirror monochromator	θ_max_ = 29.0°, θ_min_ = 2.0°
Detector resolution: 5.81 pixels mm^-1^	*h* = −13→13
rotation method, ω scans	*k* = −14→15
29260 measured reflections	*l* = −15→14
5839 independent reflections	

### 2-(3,4,5-Triphenylphenyl)acetic acid acetonitrile monosolvate Refinement

**Table:**

Refinement on *F*^2^	Primary atom site location: dual
Least-squares matrix: full	Hydrogen site location: mixed
*R*[*F*^2^ > 2σ(*F*^2^)] = 0.040	H atoms treated by a mixture of independent and constrained refinement
*wR*(*F*^2^) = 0.106	*w* = 1/[σ^2^(*F*_o_^2^) + (0.0493*P*)^2^ + 0.1858*P*] where *P* = (*F*_o_^2^ + 2*F*_c_^2^)/3
*S* = 1.07	(Δ/σ)_max_ < 0.001
5839 reflections	Δρ_max_ = 0.29 e Å^−^^3^
286 parameters	Δρ_min_ = −0.23 e Å^−^^3^
0 restraints	

### 2-(3,4,5-Triphenylphenyl)acetic acid acetonitrile monosolvate Special details

**Table:**

Geometry. All esds (except the esd in the dihedral angle between two l.s. planes) are estimated using the full covariance matrix. The cell esds are taken into account individually in the estimation of esds in distances, angles and torsion angles; correlations between esds in cell parameters are only used when they are defined by crystal symmetry. An approximate (isotropic) treatment of cell esds is used for estimating esds involving l.s. planes.

### 2-(3,4,5-Triphenylphenyl)acetic acid acetonitrile monosolvate Fractional atomic coordinates and isotropic or equivalent isotropic displacement parameters (Å^2^)

**Table:**

	*x*	*y*	*z*	*U*_iso_*/*U*_eq_	Occ. (<1)
O1	−0.07298 (9)	0.05965 (8)	0.36283 (9)	0.0358 (2)	
H1	−0.091 (2)	−0.0091 (19)	0.399 (2)	0.078 (6)*	
O2	0.13280 (9)	0.12762 (8)	0.55908 (8)	0.03183 (19)	
C1	0.22564 (11)	0.34842 (10)	0.52186 (10)	0.0216 (2)	
C2	0.36906 (12)	0.33544 (10)	0.54368 (10)	0.0218 (2)	
H2	0.382286	0.263815	0.483487	0.026*	
C3	0.49456 (11)	0.42462 (10)	0.65156 (10)	0.0201 (2)	
C4	0.47611 (11)	0.53195 (10)	0.74027 (10)	0.0193 (2)	
C5	0.33056 (11)	0.54609 (10)	0.71808 (10)	0.0201 (2)	
C6	0.20807 (11)	0.45380 (10)	0.60987 (10)	0.0216 (2)	
H6	0.110351	0.463520	0.596275	0.026*	
C7	0.09294 (12)	0.24659 (10)	0.40881 (11)	0.0242 (2)	
H7A	0.114936	0.210270	0.331366	0.029*	
H7B	0.006185	0.286815	0.378211	0.029*	
C8	0.05351 (11)	0.13879 (10)	0.45171 (10)	0.0224 (2)	
C9	0.64313 (11)	0.39716 (10)	0.66840 (11)	0.0211 (2)	
C10	0.66910 (12)	0.35689 (11)	0.55507 (11)	0.0259 (2)	
H10	0.594934	0.353617	0.469893	0.031*	
C11	0.80157 (13)	0.32161 (12)	0.56508 (12)	0.0301 (2)	
H11	0.817483	0.294114	0.486928	0.036*	
C12	0.91069 (13)	0.32630 (12)	0.68844 (12)	0.0290 (2)	
H12	1.001067	0.301151	0.695058	0.035*	
C13	0.88762 (12)	0.36781 (11)	0.80227 (12)	0.0274 (2)	
H13	0.963120	0.372551	0.887470	0.033*	
C14	0.75473 (12)	0.40252 (11)	0.79249 (11)	0.0244 (2)	
H14	0.739576	0.430200	0.871045	0.029*	
C15	0.60663 (11)	0.63167 (10)	0.85467 (10)	0.0208 (2)	
C16	0.70629 (12)	0.70122 (10)	0.83003 (11)	0.0249 (2)	
H16	0.692565	0.683040	0.739973	0.030*	
C17	0.82536 (12)	0.79678 (11)	0.93604 (13)	0.0305 (2)	
H17	0.891780	0.844448	0.918032	0.037*	
C18	0.84776 (13)	0.82292 (11)	1.06779 (13)	0.0334 (3)	
H18	0.929920	0.887786	1.140284	0.040*	
C19	0.74974 (13)	0.75402 (12)	1.09360 (12)	0.0315 (3)	
H19	0.764585	0.771875	1.183930	0.038*	
C20	0.63008 (12)	0.65911 (11)	0.98772 (11)	0.0255 (2)	
H20	0.563380	0.612290	1.006140	0.031*	
C21	0.30036 (11)	0.65849 (10)	0.80381 (10)	0.0211 (2)	
C22	0.35417 (12)	0.78503 (10)	0.82192 (11)	0.0246 (2)	
H22	0.416416	0.801271	0.782487	0.029*	
C23	0.31780 (13)	0.88733 (11)	0.89682 (12)	0.0293 (2)	
H23	0.354078	0.973106	0.907495	0.035*	
C24	0.22888 (14)	0.86499 (12)	0.95610 (12)	0.0337 (3)	
H24	0.204717	0.935273	1.008130	0.040*	
C25	0.17528 (15)	0.74009 (13)	0.93941 (13)	0.0354 (3)	
H25	0.114454	0.724663	0.980389	0.042*	
C26	0.20969 (13)	0.63725 (11)	0.86329 (12)	0.0280 (2)	
H26	0.171302	0.551625	0.851468	0.034*	
N1	0.7111 (2)	−0.01405 (16)	0.68515 (17)	0.0758 (5)	
C27	0.6123 (2)	0.02633 (15)	0.68979 (15)	0.0529 (4)	
C28	0.4872 (2)	0.07793 (19)	0.6979 (2)	0.0655 (5)	
H28A	0.406195	0.007112	0.673936	0.098*	0.51 (2)
H28B	0.452431	0.124632	0.634864	0.098*	0.51 (2)
H28C	0.518164	0.137051	0.790284	0.098*	0.51 (2)
H28D	0.399355	0.041271	0.609405	0.098*	0.49 (2)
H28E	0.511169	0.171985	0.724289	0.098*	0.49 (2)
H28F	0.466266	0.055539	0.765390	0.098*	0.49 (2)

### 2-(3,4,5-Triphenylphenyl)acetic acid acetonitrile monosolvate Atomic displacement parameters (Å^2^)

**Table:**

	*U*^11^	*U*^22^	*U*^33^	*U*^12^	*U*^13^	*U*^23^
O1	0.0314 (4)	0.0319 (5)	0.0287 (4)	−0.0063 (4)	0.0000 (4)	0.0126 (4)
O2	0.0300 (4)	0.0307 (4)	0.0248 (4)	−0.0032 (3)	0.0037 (3)	0.0110 (3)
C1	0.0221 (5)	0.0215 (5)	0.0199 (5)	0.0024 (4)	0.0080 (4)	0.0084 (4)
C2	0.0253 (5)	0.0207 (5)	0.0206 (5)	0.0059 (4)	0.0113 (4)	0.0073 (4)
C3	0.0210 (5)	0.0220 (5)	0.0203 (5)	0.0063 (4)	0.0105 (4)	0.0096 (4)
C4	0.0205 (5)	0.0206 (5)	0.0186 (5)	0.0048 (4)	0.0094 (4)	0.0082 (4)
C5	0.0213 (5)	0.0210 (5)	0.0211 (5)	0.0056 (4)	0.0111 (4)	0.0092 (4)
C6	0.0192 (5)	0.0236 (5)	0.0239 (5)	0.0049 (4)	0.0103 (4)	0.0099 (4)
C7	0.0229 (5)	0.0248 (5)	0.0207 (5)	0.0025 (4)	0.0071 (4)	0.0067 (4)
C8	0.0216 (5)	0.0218 (5)	0.0194 (5)	0.0044 (4)	0.0080 (4)	0.0030 (4)
C9	0.0213 (5)	0.0193 (5)	0.0245 (5)	0.0056 (4)	0.0114 (4)	0.0085 (4)
C10	0.0263 (5)	0.0320 (6)	0.0233 (5)	0.0104 (4)	0.0127 (4)	0.0121 (5)
C11	0.0318 (6)	0.0373 (6)	0.0308 (6)	0.0144 (5)	0.0204 (5)	0.0142 (5)
C12	0.0251 (5)	0.0343 (6)	0.0358 (6)	0.0136 (5)	0.0173 (5)	0.0166 (5)
C13	0.0239 (5)	0.0321 (6)	0.0272 (6)	0.0099 (4)	0.0099 (4)	0.0132 (5)
C14	0.0260 (5)	0.0272 (5)	0.0234 (5)	0.0090 (4)	0.0129 (4)	0.0100 (4)
C15	0.0192 (5)	0.0203 (5)	0.0212 (5)	0.0072 (4)	0.0075 (4)	0.0067 (4)
C16	0.0229 (5)	0.0239 (5)	0.0271 (5)	0.0064 (4)	0.0112 (4)	0.0076 (4)
C17	0.0228 (5)	0.0243 (5)	0.0396 (7)	0.0050 (4)	0.0121 (5)	0.0076 (5)
C18	0.0236 (5)	0.0259 (6)	0.0331 (6)	0.0069 (5)	0.0034 (5)	−0.0001 (5)
C19	0.0307 (6)	0.0340 (6)	0.0207 (5)	0.0142 (5)	0.0064 (5)	0.0035 (5)
C20	0.0252 (5)	0.0289 (6)	0.0226 (5)	0.0108 (4)	0.0102 (4)	0.0091 (4)
C21	0.0190 (5)	0.0239 (5)	0.0195 (5)	0.0072 (4)	0.0075 (4)	0.0075 (4)
C22	0.0211 (5)	0.0256 (5)	0.0254 (5)	0.0059 (4)	0.0091 (4)	0.0087 (4)
C23	0.0282 (6)	0.0242 (5)	0.0281 (6)	0.0088 (4)	0.0072 (5)	0.0067 (4)
C24	0.0404 (7)	0.0368 (6)	0.0260 (6)	0.0227 (5)	0.0156 (5)	0.0098 (5)
C25	0.0440 (7)	0.0448 (7)	0.0365 (7)	0.0239 (6)	0.0290 (6)	0.0212 (6)
C26	0.0317 (6)	0.0301 (6)	0.0320 (6)	0.0125 (5)	0.0195 (5)	0.0153 (5)
N1	0.0926 (12)	0.0596 (10)	0.0610 (10)	0.0290 (9)	0.0321 (9)	0.0031 (8)
C27	0.0676 (11)	0.0396 (8)	0.0378 (8)	0.0121 (8)	0.0141 (8)	0.0089 (6)
C28	0.0614 (11)	0.0703 (12)	0.0682 (12)	0.0228 (9)	0.0213 (9)	0.0390 (10)

### 2-(3,4,5-Triphenylphenyl)acetic acid acetonitrile monosolvate Geometric parameters (Å, º)

**Table:**

O1—H1	1.01 (2)	C15—C20	1.3928 (15)
O1—C8	1.3022 (13)	C16—H16	0.9500
O2—C8	1.2196 (13)	C16—C17	1.3878 (16)
C1—C2	1.3862 (14)	C17—H17	0.9500
C1—C6	1.3848 (15)	C17—C18	1.3831 (18)
C1—C7	1.5063 (14)	C18—H18	0.9500
C2—H2	0.9500	C18—C19	1.3869 (18)
C2—C3	1.3955 (15)	C19—H19	0.9500
C3—C4	1.4074 (14)	C19—C20	1.3869 (16)
C3—C9	1.4926 (14)	C20—H20	0.9500
C4—C5	1.4091 (13)	C21—C22	1.3938 (15)
C4—C15	1.4906 (14)	C21—C26	1.3956 (15)
C5—C6	1.3959 (14)	C22—H22	0.9500
C5—C21	1.4869 (14)	C22—C23	1.3850 (16)
C6—H6	0.9500	C23—H23	0.9500
C7—H7A	0.9900	C23—C24	1.3823 (17)
C7—H7B	0.9900	C24—H24	0.9500
C7—C8	1.5111 (15)	C24—C25	1.3811 (19)
C9—C10	1.3950 (15)	C25—H25	0.9500
C9—C14	1.3951 (15)	C25—C26	1.3846 (16)
C10—H10	0.9500	C26—H26	0.9500
C10—C11	1.3849 (15)	N1—C27	1.134 (2)
C11—H11	0.9500	C27—C28	1.445 (3)
C11—C12	1.3823 (16)	C28—H28A	0.9800
C12—H12	0.9500	C28—H28B	0.9800
C12—C13	1.3840 (16)	C28—H28C	0.9800
C13—H13	0.9500	C28—H28D	0.9800
C13—C14	1.3873 (15)	C28—H28E	0.9800
C14—H14	0.9500	C28—H28F	0.9800
C15—C16	1.3946 (15)		
			
C8—O1—H1	108.3 (11)	C20—C15—C16	118.67 (10)
C2—C1—C7	120.40 (9)	C15—C16—H16	119.7
C6—C1—C2	118.42 (9)	C17—C16—C15	120.52 (11)
C6—C1—C7	121.11 (9)	C17—C16—H16	119.7
C1—C2—H2	119.1	C16—C17—H17	119.9
C1—C2—C3	121.86 (10)	C18—C17—C16	120.29 (11)
C3—C2—H2	119.1	C18—C17—H17	119.9
C2—C3—C4	119.49 (9)	C17—C18—H18	120.1
C2—C3—C9	116.97 (9)	C17—C18—C19	119.70 (11)
C4—C3—C9	123.51 (9)	C19—C18—H18	120.1
C3—C4—C5	118.89 (9)	C18—C19—H19	120.0
C3—C4—C15	121.46 (9)	C20—C19—C18	120.10 (11)
C5—C4—C15	119.64 (9)	C20—C19—H19	120.0
C4—C5—C21	122.60 (9)	C15—C20—H20	119.6
C6—C5—C4	119.77 (9)	C19—C20—C15	120.72 (11)
C6—C5—C21	117.61 (9)	C19—C20—H20	119.6
C1—C6—C5	121.55 (9)	C22—C21—C5	122.06 (9)
C1—C6—H6	119.2	C22—C21—C26	118.55 (10)
C5—C6—H6	119.2	C26—C21—C5	119.32 (9)
C1—C7—H7A	109.2	C21—C22—H22	119.7
C1—C7—H7B	109.2	C23—C22—C21	120.66 (10)
C1—C7—C8	112.18 (8)	C23—C22—H22	119.7
H7A—C7—H7B	107.9	C22—C23—H23	119.9
C8—C7—H7A	109.2	C24—C23—C22	120.18 (11)
C8—C7—H7B	109.2	C24—C23—H23	119.9
O1—C8—C7	113.30 (9)	C23—C24—H24	120.1
O2—C8—O1	123.66 (10)	C25—C24—C23	119.75 (11)
O2—C8—C7	123.04 (10)	C25—C24—H24	120.1
C10—C9—C3	119.25 (9)	C24—C25—H25	119.8
C10—C9—C14	118.32 (9)	C24—C25—C26	120.38 (11)
C14—C9—C3	122.30 (9)	C26—C25—H25	119.8
C9—C10—H10	119.6	C21—C26—H26	119.8
C11—C10—C9	120.88 (10)	C25—C26—C21	120.47 (11)
C11—C10—H10	119.6	C25—C26—H26	119.8
C10—C11—H11	119.9	N1—C27—C28	179.11 (18)
C12—C11—C10	120.20 (10)	C27—C28—H28A	109.5
C12—C11—H11	119.9	C27—C28—H28B	109.5
C11—C12—H12	120.2	C27—C28—H28C	109.5
C11—C12—C13	119.67 (10)	C27—C28—H28D	109.5
C13—C12—H12	120.2	C27—C28—H28E	109.5
C12—C13—H13	119.9	C27—C28—H28F	109.5
C12—C13—C14	120.29 (10)	H28A—C28—H28B	109.5
C14—C13—H13	119.9	H28A—C28—H28C	109.5
C9—C14—H14	119.7	H28B—C28—H28C	109.5
C13—C14—C9	120.63 (10)	H28D—C28—H28E	109.5
C13—C14—H14	119.7	H28D—C28—H28F	109.5
C16—C15—C4	120.43 (9)	H28E—C28—H28F	109.5
C20—C15—C4	120.89 (9)		
			
C1—C2—C3—C4	−0.81 (14)	C6—C1—C7—C8	−90.31 (11)
C1—C2—C3—C9	177.24 (9)	C6—C5—C21—C22	−121.95 (11)
C1—C7—C8—O1	170.53 (9)	C6—C5—C21—C26	55.02 (13)
C1—C7—C8—O2	−9.46 (15)	C7—C1—C2—C3	−176.72 (9)
C2—C1—C6—C5	0.35 (14)	C7—C1—C6—C5	177.49 (9)
C2—C1—C7—C8	86.78 (12)	C9—C3—C4—C5	−177.54 (9)
C2—C3—C4—C5	0.38 (14)	C9—C3—C4—C15	3.78 (14)
C2—C3—C4—C15	−178.30 (9)	C9—C10—C11—C12	−0.21 (18)
C2—C3—C9—C10	44.96 (13)	C10—C9—C14—C13	−0.39 (16)
C2—C3—C9—C14	−130.96 (11)	C10—C11—C12—C13	−0.72 (18)
C3—C4—C5—C6	0.38 (14)	C11—C12—C13—C14	1.09 (18)
C3—C4—C5—C21	−177.95 (9)	C12—C13—C14—C9	−0.53 (17)
C3—C4—C15—C16	59.69 (13)	C14—C9—C10—C11	0.77 (16)
C3—C4—C15—C20	−121.55 (11)	C15—C4—C5—C6	179.09 (9)
C3—C9—C10—C11	−175.31 (10)	C15—C4—C5—C21	0.76 (14)
C3—C9—C14—C13	175.56 (10)	C15—C16—C17—C18	0.91 (16)
C4—C3—C9—C10	−137.08 (10)	C16—C15—C20—C19	0.27 (15)
C4—C3—C9—C14	47.01 (14)	C16—C17—C18—C19	−0.63 (17)
C4—C5—C6—C1	−0.76 (15)	C17—C18—C19—C20	0.18 (17)
C4—C5—C21—C22	56.41 (14)	C18—C19—C20—C15	0.00 (16)
C4—C5—C21—C26	−126.62 (11)	C20—C15—C16—C17	−0.72 (15)
C4—C15—C16—C17	178.07 (9)	C21—C5—C6—C1	177.65 (9)
C4—C15—C20—C19	−178.51 (10)	C21—C22—C23—C24	0.82 (16)
C5—C4—C15—C16	−118.98 (11)	C22—C21—C26—C25	−0.50 (16)
C5—C4—C15—C20	59.77 (13)	C22—C23—C24—C25	−0.56 (17)
C5—C21—C22—C23	176.70 (10)	C23—C24—C25—C26	−0.23 (19)
C5—C21—C26—C25	−177.58 (10)	C24—C25—C26—C21	0.76 (18)
C6—C1—C2—C3	0.45 (14)	C26—C21—C22—C23	−0.29 (15)

### 2-(3,4,5-Triphenylphenyl)acetic acid acetonitrile monosolvate Hydrogen-bond geometry (Å, º)

**Table:**

*D*—H···*A*	*D*—H	H···*A*	*D*···*A*	*D*—H···*A*
O1—H1···O2^i^	1.01 (2)	1.62 (2)	2.624 (1)	174 (2)

Symmetry code: (i) −*x*, −*y*, −*z*+1.
